# Unification of behavioural, computational and neural accounts of word production errors in post-stroke aphasia

**DOI:** 10.1016/j.nicl.2018.03.031

**Published:** 2018-03-27

**Authors:** Marija Tochadse, Ajay D. Halai, Matthew A. Lambon Ralph, Stefanie Abel

**Affiliations:** a Neuroscience and Aphasia Research Unit, University of Manchester, United Kingdom; b Department of Psychology, Philipps University of Marburg, Germany

**Keywords:** Semantics, Phonology, Principal component analysis, Cognitive model, Chronic aphasia, Naming errors

## Abstract

Neuropsychological assessment, brain imaging and computational modelling have augmented our understanding of the multifaceted functional deficits in people with language disorders after stroke. Despite the volume of research using each technique, no studies have attempted to assimilate all three approaches in order to generate a unified behavioural-computational-neural model of post-stroke aphasia.

The present study included data from 53 participants with chronic post-stroke aphasia and merged: aphasiological profiles based on a detailed neuropsychological assessment battery which was analysed with principal component and correlational analyses; measures of the impairment taken from Dell's computational model of word production; and the neural correlates of both behavioural and computational accounts analysed by voxel-based correlational methodology.

As a result, all three strands coincide with the separation of semantic and phonological stages of aphasic naming, revealing the prominence of these dimensions for the explanation of aphasic performance. Over and above three previously described principal components (phonological ability, semantic ability, executive-demand), we observed auditory working memory as a novel factor. While the phonological Dell parameter was uniquely related to phonological errors/factor, the semantic parameter was less clear-cut, being related to both semantic errors and omissions, and loading heavily with semantic ability and auditory working memory factors. The close relationship between the semantic Dell parameter and omission errors recurred in their high lesion-correlate overlap in the anterior middle temporal gyrus. In addition, the simultaneous overlap of the lesion correlate of omission errors with more dorsal temporal regions, associated with the phonological parameter, highlights the multiple drivers that underpin this error type. The novel auditory working memory factor was located along left superior/middle temporal gyrus and ventral inferior parietal lobe.

The present study fused computational, behavioural and neural data to gain comprehensive insights into the nature of the multifaceted presentations in aphasia. Our unified account contributes enhanced knowledge on dimensions explaining chronic post-stroke aphasia, the variety of factors affecting inter-individual variability, the neural basis of performance, and potential clinical implications.

## Highlights

• A unified approach converging computational, behavioural and neural data is generated.
• Semantics/phonology are identified as most prominent dimensions of chronic post-stroke aphasia.
• Inter-individual language variability is affected by executive processing and auditory working memory.
• Omission errors are predominantly associated with semantically-related brain regions.
• Previously-neglected omission errors occur frequently and hold high diagnostic value.

## 1 Introduction

Behavioural assessment and computational modelling are important tools to understand the diverse patterns of impaired performance in people with aphasia (PWA) (Basilakos et al., 2014; Rogalsky et al., 2015; Ueno et al., 2011; Walker and Hickok, 2016; for a review see Cahana-Amitay and Albert, 2015). More recently, each approach has been linked with brain lesion data to investigate the neural basis of aphasia. Thus, the computational parameters of the Dell model (Dell et al., 2013; Schwartz et al., 2012) or behavioural assessment results (Butler et al., 2014; Halai et al., 2017; Mirman et al., 2015a, Mirman et al., 2015b) have been associated with distinct regions in the brain. However, to date there has been no attempt to unify behavioural, computational and neuroimaging data in order to gain a more comprehensive, multi-level understanding of aphasia. Therefore, the purpose of the present study was to converge: (i) the principal components of aphasic performance based on behavioural data; (ii) measures of the impairment taken from a computational model of aphasic naming; and, (iii) the neural correlates of both behavioural and computational factors. We present principal component and correlational analyses of data from a large neuropsychological assessment battery and from computer simulations in the Dell interactive two-step model of word production (Abel et al., 2009; Dell et al., 1997, Dell et al., 2013; Foygel and Dell, 2000; Schwartz et al., 2006), with subsequent mapping of the model parameters and behavioural PCA components onto the brain using voxel-based correlational methodology (VBCM: Basilakos et al., 2014; Butler et al., 2014; Halai et al., 2017; Tyler et al., 2005). By merging these three levels of description from behavioural, computational and neuroimaging disciplines, we offer converging evidence on the theoretical and neural bases of the variety of behavioural presentations in aphasia.

### 1.1 Interactive two-step model

**Fig. 1:**
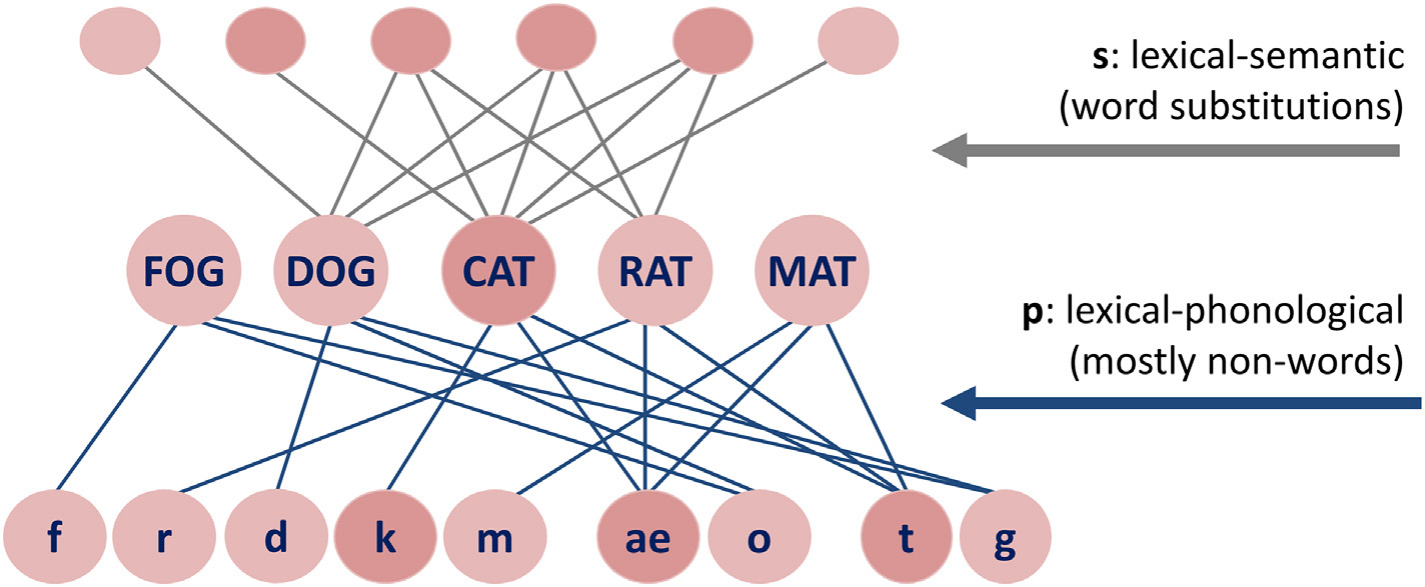
Impairment types in the Dell model (Foygel and Dell, 2000). The model includes a semantic feature layer, an intermediate lexical layer with word entries, and a phonological layer. Nodes of neighbouring layers are bi-directionally connected. The model features a two-stage access of lexical and then phonological entries, which occurs via spreading activation along the connections in the network.

In accordance with other models of word production (see overview in Rapp and Goldrick, 2006), Dell's interactive two-step model of word production (Foygel and Dell, 2000) assumed lexical functions to be split into semantic and phonological processes. The cognitive model aimed to explain intact and impaired performance in confrontation naming. It contained three layers of interconnected nodes as shown in Fig. 1, namely semantic feature nodes depicted at the top, lexical nodes in the middle, and phonological nodes at the bottom. Naming occurred in two retrieval steps: first, lexical retrieval through activation spreading from semantic feature nodes to lexical nodes; and second, phonological retrieval through activation spreading from lexical to phonological nodes. The flow of activation between layers was interactive, spreading along bidirectional connections between neighbouring layers, and it was modulated by the weights of lexical-semantic connections (*s*) and lexical-phonological connections (*p*), respectively. The model explained naming errors in aphasic speakers by attributing the impairment to reduced semantic and/or phonological weights, with the former being broadly associated with word errors and the latter with mainly non-words. Thus, smaller parameter weights indicated greater impairment.

A recent paper by Dell et al. (2013), suggested that the model parameters include more processes than previously assumed, drawing their conclusion from regression analyses of behavioural data and voxel-based lesion-parameter mapping (VLPM). As in voxel-lesion symptom mapping (VLSM: Bates et al., 2003), VLPM attempts to relate the variation in model parameters for each patient to the status of voxels across the brain (intact or lesioned). While in earlier versions of the Dell model the parameters were thought to be restricted to connection weights within the lexicon only, current understanding assumes that the *s* parameter represents semantic representations and semantic control processes over and above the lexical-semantic weight. The *p* parameter includes phonological representations and aspects of articulation over and above the lexical-phonological weight. Walker and Hickok (2016) recently provided a new fitting algorithm and website (http://www.cogsci.uci.edu/~alns/webfit) for the SP-model by Foygel and Dell (2000). It constrains parameter values to be below a presumably normal level, and thereby provides an improved fit.

### 1.2 Principal component analysis

Recent studies have demonstrated the separation of semantic, phonological and other cognitive processes in aphasic performance by use of varimax rotated principal component analysis (PCA) (Butler et al., 2014; Halai et al., 2017; Lambon Ralph et al., 2002; Mirman et al., 2015a, Mirman et al., 2015b). PCA can be used as a useful exploratory tool as it can extract the components that underlie a set of correlated variables (e.g., the latent structure underlying a large neuropsychological battery). To do so, variance in the variables is first redistributed across an equal number of components as there are variables. In a second step, a predefined criterion is used to extract only as many components as necessary to explain a ‘sufficient’ amount of variance. Components can then be rotated, which allows clearer cognitive interpretation of the components while maintaining their orthogonality. While it is possible to allow oblique rotation of components, maintaining orthogonality in this investigation is useful for at least two reasons. First, a number of computational models have been developed for the language domain, with independent processes/layers representing fundamentally independent processes such as phonology, semantics and speech output (e.g., Dell et al., 2013; Ueno et al., 2011). In addition, co-linearity in neuroimaging analyses is problematic when mapping behaviours to the brain, as the shared variance is partitioned out and the model estimates parameters based on the remaining variance, which can be noisy. As neuropsychological data are typically highly co-linear, a method to orthogonalise the data (such as PCA) has been shown to be useful in producing more interpretable neuroimaging results (see Butler et al., 2014).

Butler et al. (2014) and Halai et al. (2017) investigated the components that contribute to performance of people with aphasia (PWA) on neuropsychological tasks that involve cognitive and language functions. Along with a phonological and a semantic factor, the two studies have shown executive processing to contribute to aphasic performance. In the follow up study, Halai et al. (2017) found that speech fluency also emerged as a statistically independent factor in addition to phonology, semantics and executive function. Using a similar methodology, Mirman et al., 2015a, Mirman et al., 2015b investigated semantic and phonological error rates in the context of a wide language test battery and found four factors that were assumed to reflect a division of the language system into semantic versus phonological processes, and recognition versus production. Of these four factors identified, semantic recognition and speech production can be related to semantic and phonological factors mentioned above (Butler et al., 2014; Halai et al., 2017), respectively. Interestingly, while phonological error rate aligned with speech production, semantic errors did not load strongly on any of the first three factors but formed an independent fourth factor with only small loadings for the other assessments, indicating that they are relatively independent of the other factors.

One can pose the question as to how the *s* and *p* parameters from the Dell model relate to the PCA factors found to be crucial in describing aphasic performance. Given that semantic errors, which are a main input for the calculation of the *s* parameter, do not align with the core factors, it is especially interesting to examine whether the *s* parameter will behave in a similar way. Another point of interest is the relation of the model parameters to a more detailed range of naming error categories, as certain error types like omission errors are not taken into account in the specification of the model parameters, but constitute an important part of aphasic naming errors. Thus, our investigation set out to explore the relationship of the Dell model parameters to principal components of language in PWA and to examine whether the re-interpretation of the model parameters is consistent with their relation to this behavioural data set.

### 1.3 Symptom-lesion mapping

The dissociation of semantic and phonological language functions can also be observed at the neuroanatomical level. Previous work on relating semantic and phonological error rates to neural damage in post-stroke PWA (Schwartz et al., 2009, Schwartz et al., 2012) found a prominent left anterior temporal lobe (ATL) involvement in the production of semantic errors, along with an involvement of inferior and middle frontal gyri. When relating the primary systems language factors identified by PCA to neural damage in post-stroke PWA, the left ATL again was identified as the locus of damage underlying poorer performance on semantic tasks (Butler et al., 2014; Halai et al., 2017). Likewise, the semantic error factor identified by Mirman et al., 2015a, Mirman et al., 2015b was associated with the left ATL, while the semantic recognition factor was correlated with a frontal white matter bottleneck region medial to the insula and lateral to the basal ganglia, which the researchers ascribed to executive-semantic processing (cf. Jefferies et al., 2006) and whose damage was associated with low recovery potential (cf. Abel et al., 2015). In contrast, phonological error rate was found to correlate with postcentral gyrus, insula, superior temporal and supramarginal gyrus (Schwartz et al., 2012). The phonological factor (Butler et al., 2014; Halai et al., 2017) was associated with damage to perisylvian regions, as were the speech production and speech recognition factors (Mirman et al., 2015a, Mirman et al., 2015b), which were linked to regions superior and inferior to the Sylvian fissure. The additional speech quanta factor (the amount of speech generated), observed by Halai et al. (2017), was located in left precentral gyrus, superior insula and putamen, extending medially to the caudate nucleus.

In a recent paper using voxel-lesion parameter mapping, Dell et al. (2013) investigated the neural correlates of the parameters of the interactive two-step model of word production. They found a highly distributed map of neural correlates for parameter *s*, including the ATL, middle and inferior frontal gyri, parietal-temporal junction and angular gyrus. Parameter *p* was linked to neural damage in pre- and postcentral gyrus, insula, and supramarginal gyrus.

### 1.4 Aims and hypotheses of the current study

This investigation determined how evidence from computational modelling, neuropsychological assessment and neuroimaging converge, with particular regard to the specialisation of functions in language processing. To date, investigations have explored the relationship between lesions and either aphasiological profiles or patients' parameter fits within the Dell model. In the present study, we brought these different analyses together in order to generate a unified behavioural, computational and neural account of aphasic naming performance. This was achieved by fitting the Dell model to a large group of PWA and relating the resultant *s* and *p* parameters to the PCA behavioural components and their neural correlates.

We hypothesised (i) parameter *p* to correlate with typical phonological errors such as phonemic errors and neologisms, and to load highly with the PCA component relating to patients' phonological ability. (ii) Parameter *s* was expected to correlate with a wider range of naming errors (including omissions and semantic errors) and to load either across various PCA factors (following previous work showing that semantic errors can have multiple sources: cf. Morton and Patterson, 1980; Plaut and Shallice, 1993) or form a separate factor (following Mirman et al., 2015a, Mirman et al., 2015b). In addition, we (iii) expected to find an association between parameter *p* and perisylvian regions previously found to be involved in phonological ability. Furthermore, we (iv) aimed to specify the highly distributed map for parameter *s*, expecting the resulting map to align with converging evidence for ATL involvement in semantics. Finally, we (v) aimed to differentiate and anatomically localise PCA factors of language processing, and expected to corroborate and extend previous findings on their neural correlates.

## 2 Materials and methods

### 2.1 Participants

Fifty-three PWA were recruited from local aphasia support groups, all with post-stroke (ischaemic or haemorrhagic) speech production or comprehension difficulties. The patient population is part of a database being collected at the Neuroscience and Aphasia Research Unit since 2010; as such 28 patients have been included in previously published papers (Butler et al., 2014; Halai et al., 2017). As criteria for inclusion, PWA had to be at least 12 months post-stroke at the time of scanning and assessment, native English speakers, and have normal or corrected-to-normal hearing and vision. Exclusion criteria were contraindications for MRI scanning, pre-morbid left-handedness, or more than one stroke or any other significant neurological condition. PWA were included regardless of their level of impairment or type of aphasia; however, if they could not at least attempt 50% of items they were excluded from further analysis (7 participants). The project was approved by the local ethics committee (NRES North West – Haydock, Ethics Ref No: 01/08/94) and informed consent forms were signed by all PWA prior to participation. In the various neuroimaging analyses, data from a healthy age- and education-matched control group (8 female, 11 male) were used.

### 2.2 Overview of procedures for hypothesis testing

In order to test our hypotheses, we conducted detailed correlational and principal component analyses between large-scale neuropsychological data and the parameter weights from the model. This approach allowed us to test whether the Dell model parameters are similar/dissimilar to the core language and cognitive factors observed previously to underlie aphasic performance. To achieve this aim, correlational analyses first explored how the parameter weights were related to a wide range of naming error types. It is important to note that the Dell model takes into account five error types, but there are further error types which are disregarded. Furthermore, the a priori clustering of the error types was compared with an exploratory PCA methodology in order to determine how the model parameters related to core error types. Finally, in order to determine how the model parameters related to a wider set of neuropsychological assessments, we conducted an exploratory PCA that included both *s* and *p* parameters and the language and cognitive assessments. Once we identified the independent factors that captured both the model parameters and the detailed neuropsychological tests, we mapped these factors on to the patients' lesion distributions using VBCM, a variant of voxel-lesion symptom mapping (VLSM: Bates et al., 2003) that does not require a binary classification of the intact/lesioned brain. This additional step in mapping the components to the lesion can help in validating our understanding of the nature of the components by determining how they converge with existing literature. In order to make sure the principal component analyses were robust, we conducted multiple iterations using three different extraction methodologies with an additional cross-validation analysis to determine the optimal number of components (Ballabio, 2015; Bro et al., 2008). The cross-validation approach partitions the data into 5-folds, performs a PCA on four folds, and uses this model to predict a test variable that has been left out in the holdout fold. This is repeated so that all test variables and folds are omitted once and the resultant average root mean square error (RMSE) is calculated for each PCA model (where the model is composed of *N* = 1/number of test components). The first approach used a varimax rotation on the optimal number of components, which maximises the loading for any given test on a single component, while orthogonalising the components. However, the method of orthogonalising behavioural components has been disputed (Fabrigar et al., 1999; Henson and Roberts, 2006; Park et al., 2002; Russell, 2002), since in natural behaviours inter-correlations between underlying constructs might be expected. Therefore, in a second approach we conducted the PCA using an oblique rotation (promax). Finally, in the third approach we re-ran the PCA on the behavioural data while omitting tests that are typically related to each of the components in the clinic. We then performed post hoc correlations in order to determine if the PCA components obtained were related to the omitted tests as expected by their use in clinic. For clarity throughout the manuscript, we focus on results from the varimax rotation since this procedure allows for clear interpretations of the factors (tests maximally loading onto one factor and minimally on others) and is regularly used in our field. We report outputs from the other two methods in reference to the Supplementary materials, in order to evaluate whether they yield overlapping results.

### 2.3 Neuropsychological assessment and analysis

PWA were asked to name 64 black and white drawings from the Cambridge Naming Test (Bozeat et al., 2000) and 60 black and white drawings from the Boston Naming Test (Kaplan et al., 1983). Naming attempts were recorded and coded into one of 14 error categories, including semantic (SEM), phonemic (PHON), neologism (NEO), formal (FORM), mixed (MIX), unrelated (UNREL), initial phoneme (INITIAL), dysfluency (DSY), perseveration (PERS), circumlocution (CIRC), not-a-correct (NOT A COR), visual (VIS), omission (OM) and other (OTHER) (for descriptive details, see Table 1). Some errors occurred seldom; therefore any error category that both contributed to <2% of overall errors and was not required for computing the Dell model parameters was excluded from further PCA analyses. For each naming trial, the first complete (non-fragment) response produced within 10 s was scored; if no response was given within this time frame, the item was marked as an omission. The dependent variable for subsequent analyses was the ratio of each error type to the total number of items attempted.

**Table 1:** Re-coding of error categories.

Initial error coding: Manchester classification	Description	Re-coded Dell classification
Correct	Correct response	Correct
Dysfluency	The correct initial phonemes of the word are produced followed by a correct production of the target word	

Not-a-correct	Correct responses including an indication that the subject believes that the response was incorrect or was unsure of accuracy, or the response was posed as a question

Semantic error	A real word that was semantically related but not phonologically related to the target	Semantic

Formal error	A real word that was phonologically related to the target by the phonemic definition, but not semantically related to the target	Formal

Mixed error	A semantically and phonologically related real word, but not morphologically related	Mixed

Unrelated error	A real word that was neither semantically or phonologically related to the target	Unrelated

Phonemic error	A non-word with the correct first or last phoneme or at least two other phonemes in their correct position, or at least 30% correct phonemes in any position	Non-word

Neologism	A non-word that was phonologically unrelated to the target by the phonemic definition

Perseveration	Repetition of a previous response	Coded in relation to target

Circumlocution	A description of the target alone, without an attempt to produce the name	Omission

Omission	No attempt to produce the target	

Initial error	Only the initial phoneme of the word is produced	

Visual error	Incorrect visual identification of the target

Other errors	Could not be classified or does not fit into any of the other categories

Not-a-incorrect	Incorrect responses including an indication that the subject believes that the response was incorrect or was unsure of accuracy, or the response was posed as a question	Not re-coded

Additionally, PWA were tested on an extensive language assessment battery (described in Butler et al., 2014; Halai et al., 2017). These included subtests from the Psycholinguistic Assessments of Language Processing in Aphasia (PALPA) battery (Kay et al., 1992): auditory discrimination using non-word (PALPA1) and word minimal pairs (PALPA2); and immediate and delayed repetition of non-words (PALPA8) and words (PALPA9). Tests from the 64-item Cambridge Semantic Battery (Bozeat et al., 2000) were included: spoken and written versions of the word-to-picture matching task and Camel and Cactus Test (pictures). To increase the sensitivity to semantic deficits we used a written 96-trial synonym judgement test (Jefferies et al., 2009). The spoken sentence comprehension task from the Comprehensive Aphasia Test (CAT) (Swinburn et al., 2005) was used to assess sentential receptive skills. Speech production deficits were assessed by coding responses to the ‘Cookie theft’ picture in the Boston Diagnostic Aphasia Examination (BDAE) (Goodglass et al., 2000), which included tokens (TOK), mean length of utterance (MLU), type/token ratio (TTR) and words-per-minute (WPM). The additional cognitive tests included forward and backward digit span (Wechsler, 1987), the Brixton Spatial Anticipation Task (Burgess and Shallice, 1997), and Raven's Coloured Progressive Matrices (Raven, 1962). All scores were converted into percentages; if no maximum score was available for the test, we used the maximum score in the dataset to scale the data. Assessments were conducted with PWA over several testing sessions (range 1–8), with their pace and number determined by the participant.

### 2.4 Model fitting

Model weight parameters, *s* and *p*, were obtained from the new online WebFit program introduced by Walker and Hickok (2016) (http://www.cogsci.uci.edu/~alns/webfit). WebFit generates the best fitting parameters by feeding the model with each PWA's actual number of errors within each of these categories. The five error categories entered into WebFit were: semantic, formal, mixed, unrelated and non-word errors (containing phonemic and neologism errors). Perseverations were re-coded in relation to the target. Omissions, circumlocutions, initial, visual, morphological and other errors were not entered into WebFit, but treated as omissions according to the independence account (see Schwartz et al., 2006).

### 2.5 Correlational analysis and principal component analysis

In order to determine how similar the *s* and *p* parameters were in comparison with (a) individual error categories, (b) grouped categories derived from PCA decomposition, and (c) the core language features derived from a PCA decomposition of a large neuropsychological battery, the analysis was split into two parts. First, correlational analyses were performed (SPSS 20.0) with the square root of *s* and *p* parameter values produced by the WebFit program on the one hand for (a) each error category and (b) the grouped errors on the other hand. The grouped errors were achieved by performing a varimax rotated PCA on the naming errors (extracting components with eigenvalue >1). Consecutively (c), the square root of *s* and *p* parameters and the language assessment tests were entered simultaneously into a PCA with varimax rotation in order to examine how the model parameters relate to broader measures of language and cognitive performance. Components were identified using a cross-validation approach and then rotated while maintaining orthogonality (varimax) to allow for clear behavioural interpretation of each factor. Individual PWA scores on each extracted factor were then used as behavioural covariates in the neuroimaging analysis.

### 2.6 Acquisition of neuroimaging data

High resolution structural T1-weighted Magnetic Resonance Imaging (MRI) scans were acquired on a 3.0 Tesla Philips Achieva scanner (Philips Healthcare, Best, The Netherlands) using an 8-element SENSE head coil. A T1-weighted inversion recovery sequence with 3D acquisition was employed, with the following parameters: TR (repetition time) = 9.0 ms, TE (echo time) = 3.93 ms, flip angle = 8°, 150 contiguous slices, slice thickness = 1 mm, acquired voxel size 1 mm^3^, matrix size 256 × 256, FOV = 256 mm, TI (inversion time) = 1150 ms, SENSE acceleration factor 2.5, total scan acquisition time = 575 s.

### 2.7 Analysis of neuroimaging data

**Fig. 2:**
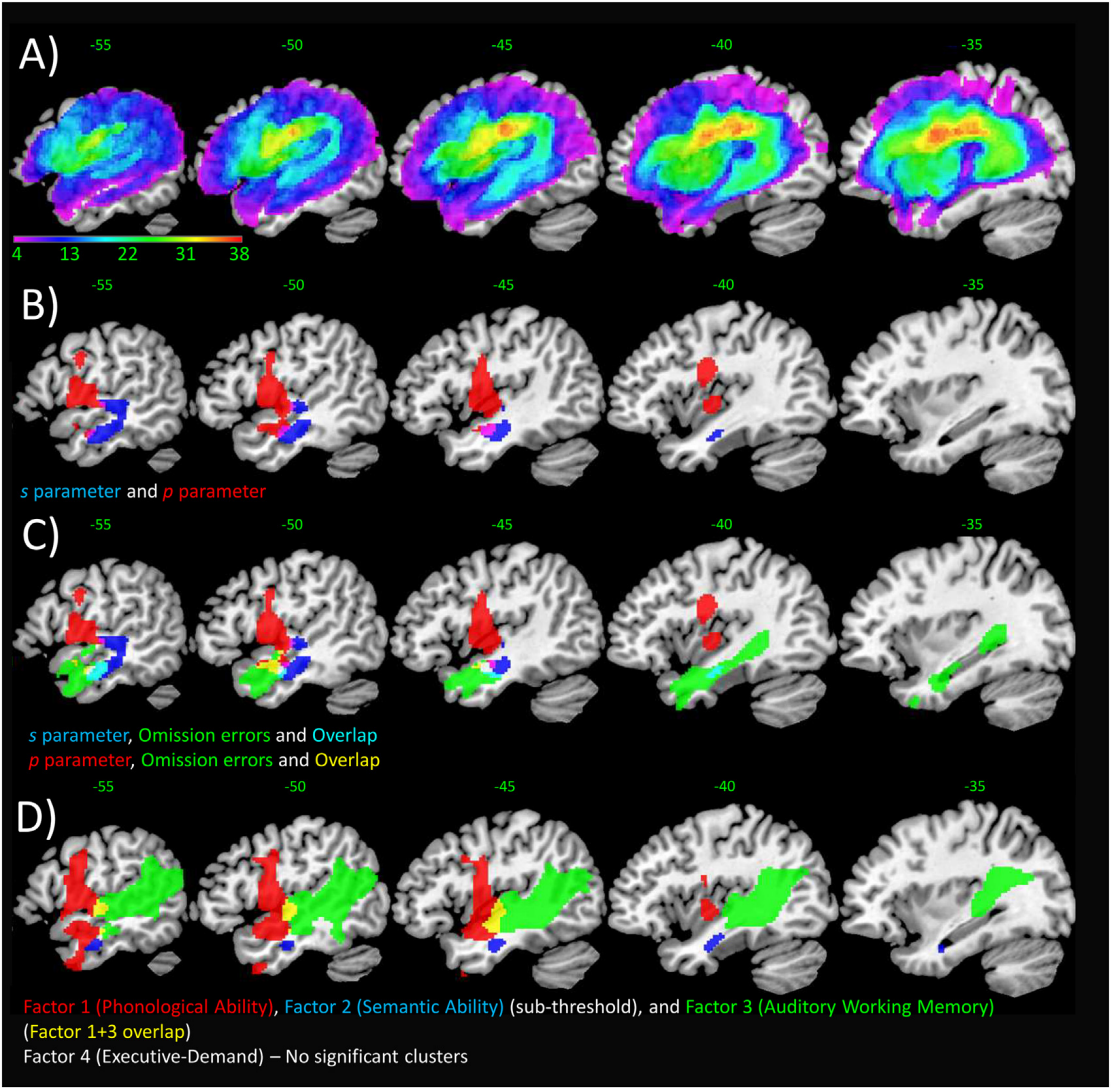
(A) Lesion overlap map across PWA in the current study. Maximum voxel = 38 (x, y, z: −21, −11, 26), located in the left superior corona radiata white matter. (B) VBCM analysis for *s* parameter weight (blue) and *p* parameter weight (red) (voxel height *p* < 0.005, FWE-cluster corrected *p* < 0.05). (C) VBCM analysis for *s* and *p* parameter weights, omission errors, and their overlap (voxel height threshold *p* = 0.005, FWE-cluster corrected *p* < 0.05). (D) VBCM correlations on factors from omnibus principal component analysis. Significant clusters are colour coded according to legend and thresholded at *p* = 0.005 voxel height, FWE-cluster corrected *p* < 0.05 (except blue cluster in D) which is at *p* = 0.005 voxel height uncorrected, 244 voxels). All results corrected for age, education and lesion volume; see Supplementary Table S4 for details on peak coordinates and anatomy. (For interpretation of the references to colour in this figure legend, the reader is referred to the web version of this article.)

Structural MRI scans were pre-processed with Statistical Parametric Mapping software (SPM8: Wellcome Trust Centre for Neuroimaging, http://www.fil.ion.ucl.ac.uk/spm/). The images were normalised into standard Montreal Neurological Institute (MNI) space using a modified unified segmentation-normalisation procedure optimised for focal lesioned brains (Seghier et al., 2008). Data from all PWA and all healthy controls were entered into the segmentation-normalisation algorithm. Images were then smoothed with an 8 mm full-width-half-maximum (FWHM) Gaussian kernel and used in the lesion analyses described below. The lesion of each PWA was automatically identified using an outlier detection algorithm, compared to age and education matched healthy controls, based on fuzzy clustering. We used the default parameters for the automated lesion identification procedure apart from the lesion definition ‘U-threshold’, which was set to 0.5 to create a binary lesion image. This was modified from 0.3 to 0.5 after comparing the results obtained from a sample of PWA to what would be nominated as lesioned tissue by an expert neurologist, and they visually inspected each image and modified the edges if necessary. The binary lesion images generated were only used to create the lesion overlap map in Fig. 2A. We selected the Seghier et al. (2008) method as it is objective and efficient for a large sample of PWA (Wilke et al., 2011), in comparison to a labour intensive hand-traced lesion mask. We should note here, explicitly, that although commonly referred to as an automated ‘lesion’ segmentation method, the technique detects areas of unexpected tissue class – and, thus, identifies missing grey and white matter but also areas of augmented CSF space.

Brain regions where tissue concentration as measured by T1-weighted signal intensity correlated with individual error measures or PCA factor scores were assessed using a voxel-based correlational methodology (VBCM: Tyler et al., 2005), a variant of voxel-lesion symptom mapping (Bates et al., 2003). VBCM does not require a binary classification of the intact/lesioned brain to be marked, as in the case of VLSM, as both the behaviour and signal intensity measures are treated as continuous variables (conducted in SPM8). As such we used the smoothed MNI normalised T1 images in subsequent VBCM analyses. Two analyses were conducted and all reported anatomical regions were located on the left hemisphere. We included age and years of education as covariates of no interest in all models. First, the neural correlates of the *s* and *p* parameters were determined separately using VBCM regression models and simultaneously using a multiple regression model. Next, factor scores of the orthogonal components obtained from a combined PCA (including parameters and language tests) were entered into the VBCM analysis. In all models, we performed analyses with and without covariates of each PWA's lesion volume (obtained using Seghier et al. (2008), lesion identification methodology). Results were generally thresholded at voxel height *p* = 0.005, FWE-cluster corrected *p* < 0.05; if no voxel exceeded threshold, we presented results at an uncorrected threshold but state this explicitly. All anatomical labels were based on the Harvard-Oxford atlas in MNI space.

## 3 Results

Summary statistics for the behavioural, demographic and model parameters can be found in Supplementary Table S1.

### 3.1 Behavioural findings

#### 3.1.1 Correlational analysis

The first analysis set out to examine the relationship between the Dell model parameters and all sub-errors. As expected from previous work (e.g., Dell et al., 2013; Schwartz et al., 2006), the *s* and *p* parameters were uncorrelated (*r* = −0.008, *p* > 0.05). As shown in Table 2, the *s* parameter was negatively correlated with semantic errors, perseverations, unrelated errors, omissions, initial errors, mixed errors, circumlocutions, and other errors, and positively with correct responses. After examining the types of perseverations, we found that they consisted mainly of semantic (49.33%) and unrelated (31.78%) errors, which mirrored the outcome of the correlation analysis for each of these categories. The *p* parameter, in contrast, was negatively correlated with phonemic, neologism, formal, and initial errors, while also being positively correlated with semantic, mixed and visual errors, as well as with correct responses including an indication that the subject believed the answer was incorrect (not-a-correct response) (Table 2).

**Table 2:** Correlation analysis of square-rooted model parameters and naming error categories.

	√*s*-weight	√*p*-weight
Correct	0.539⁎⁎⁎	0.724⁎⁎⁎
Semantic	−0.491⁎⁎⁎	0.295⁎
Perseveration	−0.711⁎⁎⁎	−0.178
Unrelated	−0.506⁎⁎⁎	−0.148
Omission	−0.467⁎⁎	−0.249
Initial	−0.440⁎⁎	−0.434⁎⁎
Mixed	−0.359⁎	0.293⁎
Circumlocution	−0.317⁎	0.167
Other	−0.437⁎⁎	−0.203
Phonemic	0.269	−0.786⁎⁎⁎
Neologism	−0.089	−0.735⁎⁎⁎
Formal	−0.111	−0.464⁎⁎
Visual	−0.059	0.362⁎
Not-a-correct	0.250	0.307⁎
Not-a-incorrect	−0.246	0.250
Dysfluency	0.137	0.169
Morphological	−0.146	0.047

Statistical thresholds:

⁎ *p* < 0.05.

⁎⁎ *p* < 0.01.

⁎⁎⁎ *p* < 0.001.

Perseverations, visual and ‘other’/miscellaneous errors were excluded from later PCA analyses due to their rare occurrence (<2% of overall errors; see Materials and Methods). The main error types of formal and unrelated errors were rare as well (1.95% and 1.56%, respectively), however as they are central to the Dell model we included them in the subsequent analyses.

The principal component analysis on the naming error data provided an exploratory approach to clustering error types; resulting in five groups of errors as listed in Table 3: 1) phonemic, formal, neologism and initial (termed PhonErr); 2) semantic, mixed and not-a-incorrect (termed SemErr); 3) dysfluency and not-a-correct (termed DysErr); 4) circumlocution errors (termed CircErr); and 5) omissions (termed OmErr). We computed the correlations between these clustered naming error factor scores and the *s* and *p* model parameters (Table 3): *s* negatively correlated with SemErr, OmErr and CircErr, which suggests that PWA exhibit increased errors of these types if they have a poor *s* parameter weight. The *p* weight negatively correlated with PhonErr and positively with SemErr (Table 3). Results from the single categories and PCA combined errors are highly overlapping, so that poor *s* weights led to more meaning-related errors or omissions, while poor *p* weights lead to more errors of sound structure.

**Table 3:** Correlation analysis of square-rooted model parameters and weighted naming error factors obtained from a PCA of error types.

	√*s*-weight	√*p*-weight
PhonErr	0.112	−0.908⁎⁎⁎
SemErr	−0.414⁎⁎	0.321⁎
DysErr	0.234	0.284
CircErr	−0.359⁎	0.242
OmErr	−0.462⁎⁎	−0.232

Principal components of error types formed five categories: phonological errors (PhonErr), semantic errors (SemErr), dysfluency errors (DysErr), circumlocution errors (CircErr) and omission errors (OmErr).

⁎ *p* < 0.05.

⁎⁎ *p* < 0.01.

⁎⁎⁎ *p* < 0.001.

#### 3.1.2 Principal component analysis

**Supplementary Fig. S1:**
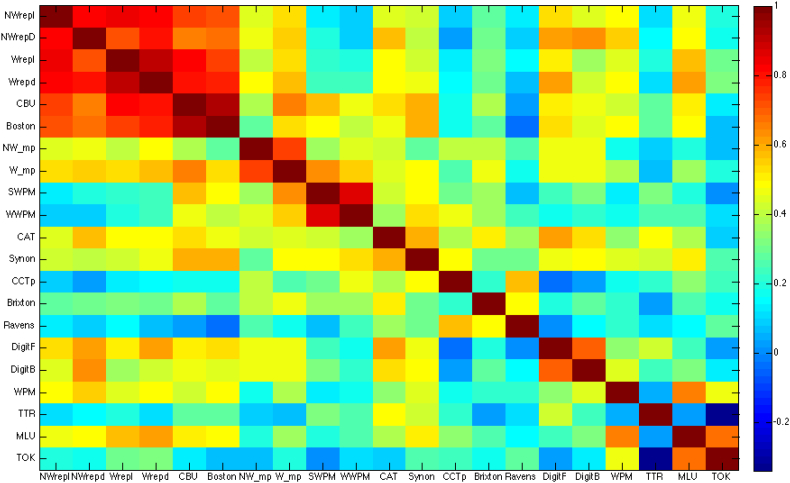
Covariance matrix of raw data converted into z-scores (therefore matrix scale is 0–1).

To examine the relationship between *s* and *p* weights and a wide range of language and cognitive tests, an omnibus PCA was performed which included 21 neuropsychological assessments and the Dell model parameters (see Supplementary Fig. S1 for covariance matrix of raw data). The cross validation analysis to determine the optimal number of components revealed that a four-component solution produced the best model that minimised RMSE. The Kaiser-Meyer-Olkin measure of sampling adequacy was 0.646 and the Bartlett's Test for Sphericity was significant (approx. chi-square = 1041.67, *df* = 253, *p* < 0.001). Three of the factors closely resembled factors reported previously (Butler et al., 2014; Halai et al., 2017) and were termed ‘phonological ability’ (29.93% variance explained by factor 1), ‘semantic ability’ (14.80% variance explained by factor 2) and ‘executive-demand’ (12.60% variance explained by factor 4). Another factor identified here was termed ‘auditory working memory’ (13.01% variance explained by factor 3), as it loaded with tasks that required the online maintenance and use of verbal inputs (forward and backwards digit span and spoken sentence comprehension).

By performing correlational analyses between the *s*/*p* weights and factors scores we observed that the *s* weight is positively correlated with semantic ability (*r* = 0.592, *p* < 0.001) and auditory working memory (*r* = 0.442, *p* = 0.002), while the *p* weight only correlated with phonological ability (*r* = 0.876, *p* < 0.001). The omnibus PCA reiterates these findings, where the *p* parameter was found to load with the phonological ability factor only and the *s* weight loaded positively across two factors, semantic ability and auditory working memory (Table 4). The results were principally identical when performing this analysis with a PCA with promax rotation (see Supplementary Table S2).

**Table 4:** Factor loadings from the omnibus principal component analysis with varimax rotation (assessments and model parameters).

	F1 29.93%	F2 14.80%	F3 13.01%	F4 12.60%	Communalities
Word repetition Del	**0.89**	0.17	0.20	0.09	0.87
Word repetition Imm	**0.88**	0.14	0.18	0.08	0.83
√***p* parameter weight**	**0.88**	0.13	−0.02	−0.01	0.79
NonWord repetition Imm	**0.86**	0.02	0.28	0.07	0.81
NonWord repetition Del	**0.80**	−0.02	0.42	0.10	0.83
Cambridge Naming Test	**0.75**	**0.55**	0.24	−0.02	0.92
Boston Naming Test	**0.74**	*0.46*	0.26	−0.07	0.84
Mean length of utterance	**0.70**	0.12	−0.19	*0.42*	0.71
Words per minute	**0.64**	−0.02	0.01	0.37	0.55
Spoken word-picture matching	0.08	**0.93**	0.17	0.13	0.91
Written word-picture matching	0.05	**0.85**	0.03	0.33	0.84
√***s* parameter weight**	0.13	**0.59**	*0.44*	−0.08	0.57
Word minimal pairs	*0.49*	**0.54**	0.21	0.27	0.64
96 Synonym judgement	0.36	*0.44*	0.33	*0.42*	0.61
Forward digit span	*0.46*	0.09	**0.74**	0.03	0.77
Type/token ratio	−0.06	0.25	**0.67**	0.02	0.51
Spoken sentence comprehension	0.31	0.23	**0.65**	*0.44*	0.76
Backward digit span	*0.41*	0.09	**0.61**	0.21	0.59
Ravens Coloured Matrices	−0.04	−0.07	0.13	**0.85**	0.74
Camel and cactus pictures	−0.03	0.33	0.00	**0.73**	0.64
Token	*0.42*	−0.04	−*0.44*	**0.52**	0.64
Brixton Spatial Anticipation	0.20	0.26	0.19	**0.52**	0.41
NonWord minimal pairs	0.31	0.28	0.34	0.38	0.43

PCA on a large neuropsychological test battery and the *s* and *p* parameter weights (square root) from the interactive two-step model (Foygel and Dell, 2000). Values ≥0.05 are indicated in bold, ≥0.04 in italics. Imm: Immediately, Del: Delayed. Numbered factors (F), with percentages showing the variance explained per factor, were termed (1) ‘phonological ability’, (2) ‘semantic ability’, (3) ‘auditory working memory’, (4) ‘executive-demand’.

As a further validity test of the PCA solution, we identified those neuropsychological tests that are used in clinical practice to determine the respective deficits. Since these highly representative tests might have driven the PCA results, we re-ran the analysis by withholding them and compared the PCA components to these typical clinical assessments of each language/cognitive domain. Thus, we removed a test of phonology (immediate non-word repetition), semantics (synonym judgement), auditory working memory (backward digit span), and executive demand (Brixton Spatial Anticipation). The resultant PCA model produced was very similar to the original PCA result. We found factors relating to phonology (Component 1), semantics (Component 2), auditory working memory (Component 3) and executive-demand (Component 4). For each component, we correlated the left out neuropsychological tests to determine the validity of the components (see Supplementary Table S3). We found that the phonology component correlated strongly with immediate non-word repetition (*r* = 0.848, *p* < 0.001) and moderately with synonym judgement (*r* = 0.415, *p* < 0.01) and backward digit span (*r* = 0.478, *p* < 0.001). Each of these three tests require good phonological ability, where it is evident that non-word repetition relies on this aspect the most, hence showing strongest correlations, while the latter tests also require some level of good phonological abilities to succeed. The semantic component correlated with synonym judgement (*r* = 0.419, *p* < 0.01), which provides supporting evidence for this factor to be related to semantics. This component was also weakly correlated with Brixton Spatial Anticipation (*r* = 0.311, *p* < 0.035), but it would not survive multiple comparison correction. The auditory working memory component only correlated with backward digit span to some extent (*r* = 0.350, *p* < 0.02), and finally the executive-demand component correlated with synonym judgement (*r* = 0.377, *p* < 0.01) and Brixton Spatial Anticipation (*r* = 0.368, *p* = 0.01). It is plausible that the synonym judgement test correlated with this component as this test is mainly used to detect semantic deficits, and it is known to demand good executive-semantic processes (cf. Jefferies et al., 2006).

### 3.2 Neuroimaging results

In order to determine the neural correlates of the model parameters and the factor scores, we performed a VBCM analysis controlling for age and education (both with and without control for lesion size). Lesion analysis for the *s* parameter revealed a significant cluster (voxel height *p* < 0.005, FWE-corrected *p* = 0.05) in anterior middle temporal gyrus (MTG) extending to anterior parahippocampal gyrus and posterior superior temporal gyrus (STG). This result survived cluster correction when lesion volume was added as a covariate. We did not find any neural correlates for the *p* parameter without lesion size correction; however, we did with lesion volume correction (voxel height *p* < 0.005, FWE-corrected *p* < 0.05) in anterior MTG/STG, Heschl's gyrus and central opercular cortex extending to insula and pre- and postcentral gyrus. In a subsequent analysis, we entered both *s* and *p* weights simultaneously and found that both the *s* weight cluster and *p* weight cluster described above survived the threshold (voxel height *p* < 0.005, FWE-corrected *p* < 0.05, corrected for lesion size) (Fig. 2B, placed below a lesion overlap map in Fig. 2A). Despite some temporal lobe overlap between both parameters, the *p* parameter was placed more anterior-dorsal compared to the *s* parameter.

Since the correlational analysis revealed that omissions were strongly related to the *s* parameter, we performed a post-hoc analysis on this special error type to investigate whether the relationship is mirrored in the lesion-symptom mapping results. Thus, we performed another VBCM which mapped the lesion correlates of omission errors (with covariates of age, education and lesion volume, FWE-corrected) in order to determine the overlap between omission errors and the *s* and *p* parameters. Omissions were associated with the MTG, partly extending to STG and inferior temporal gyrus (ITG) (Supplementary Table S4). Thus, we found a striking overlap with the *s* parameter in anterior MTG (voxel height *p* < 0.005, FWE-corrected *p* < 0.05); however, there was also overlap with the *p* parameter more dorsally in MTG/STG (see Fig. 2C).

Finally, we performed a VBCM analysis on the omnibus varimax rotated PCA factor scores to determine the neural correlates of each factor. In order to control for potential confounds, we added age and years of education as covariates as well as lesion size. Fig. 2D (Supplementary Table S4) shows significant clusters for phonological ability (red) and auditory working memory (green) (voxel height *p* < 0.005, FWE-corrected *p* < 0.05). The cluster for semantic ability did not reach significance at the current threshold, but was identified at the reduced threshold with no other spurious clusters arising (*p* = 0.005 voxel height uncorrected, 244 voxels) (blue). The executive-demand factor did not reveal significant correlations with any neural damage. The phonological ability cluster was located on anterior/middle part of middle/superior temporal gyrus (MTG/STG), planum temporale, central opercular cortex and precentral gyrus. Auditory working memory overlapped with phonological ability in the planum temporale, but the cluster extended posteriorly along Heschl's gyrus, posterior STG and posterior MTG, temporo-occipital MTG and ventral portions of the angular and supramarginal gyrus. The sub-threshold cluster identified for semantic ability was located within anterior and inferior portion of MTG, extending medially through small portions of the anterior inferior temporal gyrus, fusiform gyrus and parahippocampal gyrus as found in previous VBCM studies (Butler et al., 2014; Halai et al., 2017) and known to be involved in semantics (see review by Lambon Ralph et al., 2017).

**Supplementary Fig. S2:**
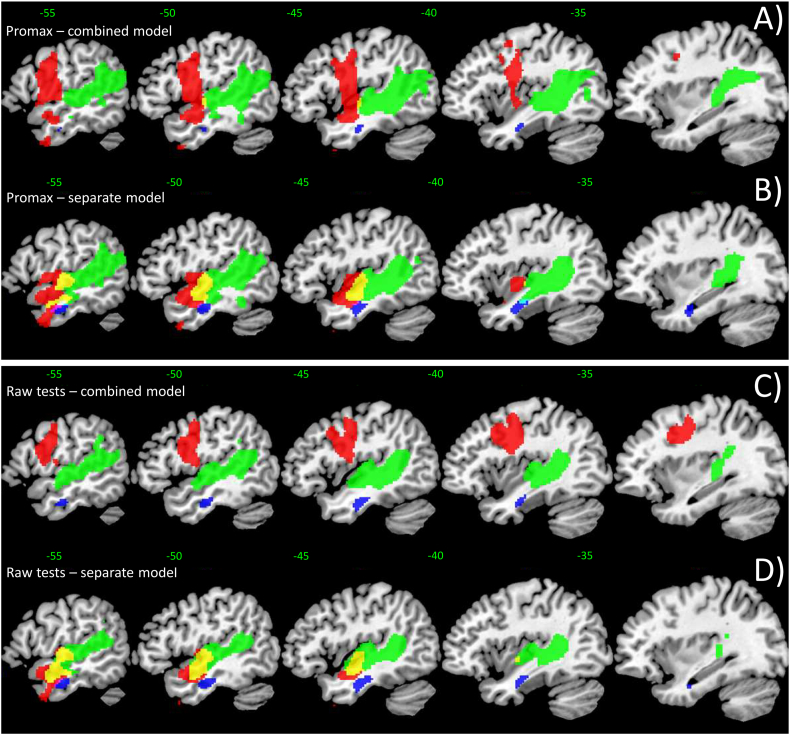
Additional analyses on the relationship of lesions and behavioural data. Factors scores from an oblique (promax rotation) principal component analysis (PCA) solution when (A) all variables are entered simultaneously into one General Linear Model, or when (B) a separate model is created for each component; and factor scores from varimax PCA when key behavioural tests (max loadings) are used (C) in a combined or (D) in a separate model. Colour-coding (A) & (B): factor 1 (phonological ability) in red, factor 2 (semantic ability) in blue (sub-threshold), factor 3 (auditory working memory) in green, and in yellow factor 1 & 3 overlap (same colour-coding as Fig. 2D). Colour-coding (C) & (D): immediate word repetition in red, spoken word-to-picture matching in blue, and forward digit span in green. Results were generally thresholded at voxel height *p* < 0.005, cluster-corrected FWE *p* < 0.05; only the sub-threshold clusters were thresholded at voxel height *p* < 0.005, voxel extent *k* > 100.

We further examined relationship between lesions and the behavioural data in two ways: 1) we used the factors scores from an oblique (promax rotation) PCA solution and 2) we used the varimax PCA model to identify key behavioural tests (max loadings), which have not been transformed into multidimensional space. The results for the promax PCA are virtually identical to the results observed using varimax PCA (Supplementary Fig. S2A), when all variables are entered simultaneously into one General Linear Model (GLM). We also created a separate model for each component to identify areas that would normally be discarded due to co-linearity, as the factors are not orthogonal – the results again were almost identical except for the phonology component, which was placed within the temporal lobe and did not extend dorsally into the precentral gyrus (Supplementary Fig. S2B). Next we chose four behavioural tests, whose loadings were high for each factor (see Table 4), to be correlated to the lesions: 1) immediate word repetition, 2) spoken word-to-picture matching, 3) forward digit span and 4) Ravens Coloured Progressive Matrices. The results revealed high overlap with already reported results (see Supplementary Fig. S2C & D). In the first instance, when all four variables were entered into a single model, the results replicated both the varimax and promax (when entered together) neural correlates (compare Fig. 1D with Supplementary Fig. S2C). Secondly, when we entered each test score as a separate model, the results replicated those found in the promax (separate model) analyses (compare Supplementary Fig. S2B with Supplementary Fig. S2D). In brief, immediate word repetition correlated with the mid/anterior parts of the middle and superior temporal gyrus (akin to phonological ability), the spoken word-to-picture matching correlated with a cluster in the middle and inferior temporal gyrus (similar to semantic ability) and forward digit span correlated with a large cluster in the mid to posterior superior temporal lobe extending posteriorly into the supramarginal gyrus (similar to auditory working memory ability).

## 4 Discussion

There are several albeit separate lines of research investigating word production errors and speech difficulties in PWA, spanning from behavioural assessment, neuroimaging and computational modelling (Basilakos et al., 2014; Rogalsky et al., 2015; Ueno et al., 2011; Walker and Hickok, 2016; for a review see Cahana-Amitay and Albert, 2015). The principal aim of this study was to draw all three key methods together for the first time – this is a key step towards generating unified theories that bridge between behavioural, computational and neural levels of explanation. Specifically, we examined the relation of the Dell computational model (Foygel and Dell, 2000) to an extensive neuropsychological test battery, as well as the neural correlates of both measures.

At the most general level, it is striking that the dissociation between semantic and phonological processes was reflected strongly across all three methodologies, suggesting that these are two of the most important dimensions that underpin aphasic performance; this was found in the Dell model (Foygel and Dell, 2000) through semantic and phonological weights and in the factors underlying aphasic performance on neuropsychological tests extracted by PCA (Butler et al., 2014; Halai et al., 2017; Mirman et al., 2015a, Mirman et al., 2015b), with a clear one-to-one mapping between these strands and to separate lesion correlates which align with independent convergent functional magnetic resonance imaging (fMRI), transcranial magnetic stimulation (TMS) and neuropsychological evidence with respect to semantic and phonological processing (Hickok and Poeppel, 2004, Hickok and Poeppel, 2007; Lambon Ralph et al., 2017; Rauschecker and Scott, 2009). These results also provide strong evidence for the validity of the extracted PCA components.

More specifically, in addition to three behavioural factors (phonology, semantic, and executive-demand) that have been previously described to underlie aphasic performance (Butler et al., 2014; Halai et al., 2017; Mirman et al., 2015a, Mirman et al., 2015b), we also obtained an additional factor - auditory working memory. This result was fairly robust as shown in the supplementary analysis (see Supplementary Table S3) where we repeated the analysis using an oblique rotated PCA in a follow up analysis where we removed a number of clinically important tests. The post-hoc correlations showed the components indeed reflected the behaviours indicated by the PCA exploration. Moreover, application of various alternative approaches to VBCM yielded similar results, corroborating the robustness of the methodological approach chosen.

In the current dataset, the ‘speech quanta’ factor, which was identified previously (Halai et al., 2017), did not materialise. Instead the words per minute and mean length of utterances loaded strongly with phonological ability as well as moderately with executive-demand. In addition, Tokens displayed a similar loading pattern, where it loaded more strongly with executive-demand but moderately with phonological ability. These results suggest that within this particular group of patients there was enough variance to subdivide input and output phonological abilities (factors 1 and 3), but in doing so the measures previously related to speech quanta typically collapsed into the output phonological abilities (factor 1) not leaving enough consistent variance to form an independent speech quanta variable; this is also supported by the neural correlates identified.

Relating the model parameters to our original factors including all tests, we found that model parameter *p* was unambiguously related to phonological errors and the phonological ability PCA factor. Following the previous literature (Mirman et al., 2015a, Mirman et al., 2015b; Morton and Patterson, 1980; Plaut and Shallice, 1993), model parameter *s* was less clear-cut, being related to both semantic errors and omissions, and loading heavily with the PCA factors of semantic ability and auditory working memory.

We also examined the relationship of the Dell model parameters and PCA factor scores to neural damage using VBCM. Interestingly, the close relationship between the *s* parameter and omission errors was mirrored by their strong lesion-correlate overlap; in addition, there was also some overlap with parts of the network for the *p* parameter, reflecting the fact that omission errors can have multiple underlying causes. Moreover, when simultaneously entering model parameters *s* and *p* and controlling for lesion size, as expected we identified a lesion cluster with maximum in anterior MTG for *s*, and a cluster in STG/central opercular cortex for *p*. In short, we were able to corroborate previous findings on neural correlates of PCA factors, and extended knowledge to our novel factor of auditory working memory. There were no significant clusters for the executive factor which also mirrors previous studies (Butler et al., 2014; Halai et al., 2017; though see Lacey et al., 2017).

### 4.1 Parameters of the Dell model: phonological weight

In the analysis of behavioural data, we confirmed and extended the findings of Schwartz et al. (2006) for the correlations of the Dell model parameters with aphasic naming errors. We found that parameter *p* was correlated negatively with errors in the phonological domain; the moderate positive correlation with semantic and mixed semantic-phonological errors can be explained by the fact that although semantics and phonology may be impaired to a different degree, PWA who are severely impaired in phonology tend to make fewer semantic errors, as the proportion of semantic errors is related to a better overall performance (see Schwartz et al., 2006) – presumably reflecting the fact that output phonology has to be sufficiently intact to be able to output semantic and other errors of commission. The same may hold for the positive correlation with visual errors, though these were rare in occurrence (<2%). Moreover, there is a positive correlation with correct responses during which the participants indicated that they believed the answer was incorrect (i.e., not-a-correct response), indicating that with better phonological competence the accurate self-monitoring of responses is more robust.

In the correlational analysis with the model parameters and the PCA error factors, *p* was correlated negatively with the PhonErr factor only, constituted by neologisms, phonemic errors, initial errors and formal errors. Likewise, when the model parameters were included in an omnibus PCA with the full background neuropsychological assessments, parameter *p* was found to load strongly yet uniquely on the phonological ability component. Both these results again highlight the notion that model parameter *p* is uniquely related to phonological abilities.

The neural correlates of the *p* parameter are in line with previous evidence for the areas within the superior temporal lobe that are involved in phonological processing. In particular, the cluster we found was located at Heschl's gyrus, which is known to process auditory input, and the surrounding areas are involved in the processing of phonological forms (Hickok and Poeppel, 2007; Mirman et al., 2015a, Mirman et al., 2015b; Rauschecker and Scott, 2009; Scott et al., 2000). Moreover, the identification of superior temporal, central opercular, insular and pre- and postcentral areas for our phonological parameter *p* using VBCM highly overlaps with regions found for phonological error frequency using VLSM by Schwartz et al. (2012), whereby it especially overlaps with their whole group result which includes persons with apraxia of speech as in our sample, and its anterior-centred focus. Moreover, it reveals high overlap with the VLSM results from Dell et al. (2013) for the *p*-parameter (as obtained in contrast to the non-lexical weight parameter of repetition *nl*, while our *p*-parameter includes both lexical and non-lexical phonology) in pre-and postcentral and insular brain areas. The STG component as found for our *p*-parameter was covered by their *nl*-parameter instead, suggesting that the STG component covers the non-lexical aspect of phonology within our data as well. The supramarginal region, as found in their analyses, has been found for our working memory factor in a more posterior region instead.

### 4.2 Parameters of the Dell model: semantic weight

Parameter *s* correlated negatively with a wide range of errors including omissions, semantic errors, perseverations, and unrelated errors. Moreover, *s* was correlated negatively with the PCA error factor OmErr (omission errors), SemErr (comprising semantic, mixed, and not-a-incorrect errors) and CircErr (comprising circumlocutions). In the omnibus PCA with neuropsychological assessments and Dell model parameters, *s* loaded positively across the factors of semantic ability and auditory working memory.

These findings indicate a higher complexity for parameter *s* than the pattern of results for the phonological parameter, *p*. Specifically, *s* is not solely linked to semantic errors or just to the underlying semantic ability neuropsychological factor. For example, s relates to omission errors to similar degree, with lower *s* weights related to greater rates of omission errors. This is particularly interesting, as omission errors are not accounted for in the Dell model (Dell et al., 2013; Foygel and Dell, 2000; Schwartz et al., 2006) and are often disregarded in studies investigating naming errors in PWA (Cloutman et al., 2009; Mirman et al., 2015a, Mirman et al., 2015b; Rohrer et al., 2008; Schwartz et al., 2009, Schwartz et al., 2012; Walker et al., 2011), although they constitute one of the most common error types in aphasia (in this sample 16% of naming responses according to our classification and 25% according to the Dell classification) and are the most common error type in semantic dementia (in which their profound word-finding difficulties are underpinned solely by progressive degradation of the semantic system: Lambon Ralph et al., 2001). Collectively, these results are consistent with accounts and models of omission errors in terms of insufficient semantic input to speech production to drive any lexical items to threshold (Laine et al., 1998; Lambon Ralph et al., 2001). Alternative theories (the lexical editor account: Baars et al., 1975; Dell et al., 2004) suggest that at least some omission errors might also relate to mechanisms beyond the speech production system which are able to monitor and edit potential responses before they are overtly generated. Our data are silent on this hypothesis, which to be tested would require an independent measure of internal speech monitoring which is not contained in our large test battery.

Notwithstanding, the omission error type is clinically significant for diagnostic purposes, and it deserves further investigation, especially regarding monitoring and editing mechanisms, since deeper understanding will probably impact on language rehabilitation. It would be important to understand whether a PWA's omission errors originate from semantic and/or phonological difficulties, in order to attribute this error type to the according origin, perform an appropriate diagnosis, and focus treatment at the appropriate level of language impairment including both linguistic and editorial processes. The current study reveals that knowledge on the neuroscience basis of this multi-faceted error type in each PWA, along with behavioural measures, might assist in determining the origin of the deficit and assigning the appropriate treatment method.

### 4.3 Lesion correlates

Regarding the lesion mapping results, we confirmed the close association of parameter *s* to semantics by identifying a specified region for *s* in the anterior MTG and anterior parahippocampal gyrus. This results is consistent with the considerable, convergent data indicating that the anterior temporal lobe (ATL) plays a major role in semantic representation (for a review see Lambon Ralph et al., 2017) as well as the importance of the ventral language route in semantic aspects of language processing (Mesulam et al., 2015; Ueno et al., 2011; Weiller et al., 2009). This cluster overlapped to a high degree with the cluster found for omission error, corroborating the behavioural findings of a close relation between parameter *s* and omission errors.

We were also able to replicate previous findings on the neural correlates of phonological ability and semantic ability (Butler et al., 2014; Halai et al., 2017; Mirman et al., 2015a, Mirman et al., 2015b), and we found neural correlates of the new auditory working memory factor in superior and middle temporal gyrus extending to ventral inferior parietal gyrus (see also Mirman et al., 2015a, Mirman et al., 2015b for a potentially related split of receptive vs. expressive spoken language), which is in accordance to the auditory-motor integration and storage phases of working memory as described by Hickok and Poeppel (2004), located in STS/superior temporal sulcus, in Sylvian parietal (Spt) area, and in inferior parietal lobe. Additionally, it overlapped with the two distinct MTG areas related to phonological ability. Interestingly, since within the temporal lobe the lesion correlates for semantic and phonological factors are located in close proximity, omission errors which were associated with the middle temporal gyrus overlapped with both factors, which explains the long-standing difficulty to unequivocally attribute omissions to either impairment type (Dell et al., 2004). In sum, the present study is a successful example of bridging between computational, behavioural and neural data to generate a unified account.

The following are the supplementary data related to this article.

Supplementary tables Image 1
