# Supplementary material for: Unification of behavioural, computational and neural accounts of word production errors in post-stroke aphasia
Source: Neuroimage Clin. 2018 Mar 27;18:952–62. doi: 10.1016/j.nicl.2018.03.031 (PMC5988441; doi:10.1016/j.nicl.2018.03.031)
Supplement: Supplementary file 1 — Supplementary tables [file mmc1.docx]

**Supplementary Table S1**. Summary statistics for 1) naming errors, 2) model parameters, 3) demographics, and the 4) neuropsychological battery

|  |  | Mean | Standard deviation | Minimum value | Maximum value |
| --- | --- | --- | --- | --- | --- |
| Naming errors | Correct | 48.43 | 25.79 | 0.81 | 90.32 |
|  | Semantic | 5.70 | 4.16 | 0 | 19.35 |
|  | Not-a-Incorrect | 2.99 | 3.55 | 0 | 16.13 |
|  | Initial | 3.23 | 4.47 | 0 | 24.36 |
|  | Neologism | 4.08 | 8.53 | 0 | 50.81 |
|  | Phonemic | 8.81 | 10.97 | 0 | 50.81 |
|  | Dysfluency | 1.88 | 2.47 | 0 | 12.10 |
|  | Not-a-correct | 1.09 | 2.07 | 0 | 8.06 |
|  | Circumlocution | 4.37 | 5.30 | 0 | 20.97 |
|  | Mixed | 2.02 | 1.51 | 0 | 5.65 |
|  | Omission | 15.88 | 16.19 | 0 | 70.97 |
|  | Formal | 1.10 | 1.77 | 0 | 8.87 |
|  | Morphological | 0.71 | 1.35 | 0 | 7.32 |
|  | Perseveration | 0.91 | 1.84 | 0 | 8.06 |
|  | Unrelated | 0.88 | 1.41 | 0 | 6.45 |
|  | Other | 0.56 | 1.59 | 0 | 9.68 |
|  | Visual | 0.33 | 0.53 | 0 | 2.42 |
| Model parameters | *√s* | 77.25 | 16.22 | 32.40 | 100 |
|  | *√p* | 71.74 | 20.99 | 5 | 100 |
| Demographics | Age (years) | 65.46 | 11.49 | 44 | 87 |
|  | Education (years) | 12.07 | 1.97 | 10 | 19 |
|  | Time post onset (months) | 54.65 | 43.28 | 4 | 192 |
|  | Lesion volume (2mm^3^ voxels) | 15620 | 10054 | 1481 | 42568 |
| Neuropsychological battery | Word repetition Immediate | 72.74 | 26.35 | 3.75 | 100 |
|  | NonWord repetition Immediate | 48.33 | 28.59 | 0 | 100 |
|  | Word repetition Delayed | 65.90 | 30.53 | 0 | 100 |
|  | Cambridge naming test | 59.31 | 28.63 | 1.56 | 96.88 |
|  | Boston naming test | 42.57 | 26.95 | 0 | 88.33 |
|  | NonWord repetition Delayed | 34.86 | 29.25 | 0 | 90 |
|  | Spoken word-picture matching | 95.59 | 8.01 | 64.06 | 100 |
|  | Written word-picture matching | 95.52 | 9.34 | 53.13 | 100 |
|  | 96 Synonym judgment | 81.52 | 10.58 | 57.29 | 96.88 |
|  | Forward digit span | 50.82 | 25.33 | 0 | 100 |
|  | Backward digit span | 28.62 | 23.00 | 0 | 100 |
|  | Spoken sentence comprehension | 68.48 | 21.45 | 12.50 | 100 |
|  | Type/Token ratio | 65.23 | 15.28 | 18.18 | 100 |
|  | Ravens Coloured Matrices | 80.74 | 15.93 | 38.89 | 100 |
|  | Camel and Cactus pictures | 82.64 | 10.55 | 51.56 | 98.44 |
|  | Token | 18.48 | 17.11 | 1.27 | 100 |
|  | Mean length of utterance | 44.72 | 22.84 | 7.13 | 100 |
|  | Words per minute | 23.36 | 18.41 | 1.30 | 100 |
|  | NonWord minimal pairs | 84.54 | 13.47 | 43.06 | 100 |
|  | Word minimal pairs | 88.13 | 11.87 | 54.17 | 100 |
|  | Brixton Spatial Anticipation | 57.47 | 14.69 | 25.45 | 88.89 |

**Supplementary Table S2.** Factor loadings from the omnibus principal component analysis with promax rotation (assessments and model parameters)

|  | F1  42.08% | F2  12.15% | F3  9.69% | F4  6.43% | Communalities |
| --- | --- | --- | --- | --- | --- |
| Word repetition Del | **0.93** | 0.37 | *0.42* | 0.25 | 0.87 |
| Word repetition Imm | **0.91** | 0.34 | 0.39 | 0.24 | 0.83 |
| NonWord repetition Imm | **0.88** | 0.23 | *0.45* | 0.21 | 0.81 |
| ***√p* parameter weight** | **0.86** | 0.28 | 0.19 | 0.15 | 0.79 |
| Cambridge naming test | **0.85** | **0.70** | **0.50** | 0.16 | 0.92 |
| NonWord repetition Del | **0.85** | 0.21 | **0.57** | 0.23 | 0.83 |
| Boston naming test | **0.82** | **0.61** | **0.50** | 0.10 | 0.84 |
| Mean length of utterance | **0.73** | 0.27 | 0.00 | **0.54** | 0.71 |
| Words per minute | **0.67** | 0.15 | 0.15 | *0.47* | 0.55 |
| NonWord minimal pairs | *0.46* | *0.44* | *0.45* | *0.46* | 0.43 |
| Spoken word-picture matching | 0.29 | **0.95** | 0.37 | 0.23 | 0.91 |
| Written word-picture matching | 0.26 | **0.88** | 0.22 | *0.42* | 0.84 |
| Word minimal pairs | **0.64** | **0.67** | *0.41* | *0.40* | 0.64 |
| ***√s* parameter weight** | 0.28 | **0.66** | **0.57** | 0.01 | 0.57 |
| 96 Synonym judgment | **0.54** | **0.61** | *0.49* | **0.52** | 0.61 |
| Forward digit span | **0.58** | 0.30 | **0.82** | 0.12 | 0.77 |
| Spoken sentence comprehension | **0.51** | *0.45* | **0.74** | **0.51** | 0.76 |
| Backward digit span | **0.54** | 0.30 | **0.69** | 0.29 | 0.59 |
| Type/Token ratio | 0.10 | 0.35 | **0.68** | 0.04 | 0.51 |
| Ravens Coloured Matrices | 0.11 | 0.07 | 0.12 | **0.82** | 0.74 |
| Camel and Cactus pictures | 0.15 | *0.41* | 0.07 | **0.74** | 0.64 |
| Token | *0.41* | 0.04 | -0.33 | **0.58** | 0.64 |
| Brixton Spatial Anticipation | 0.35 | 0.39 | 0.29 | **0.57** | 0.41 |

PCA on a large neuropsychological test battery and the *s* and *p* parameter weights (square root) from the interactive two-step model (Foygel & Dell, 2000). Values ≥ 0.05 are indicated in bold, ≥ 0.04 in italics. Imm: Immediately, Del: Delayed. Numbered factors (F) with percentages showing the variance explained per factor.

**Supplementary Table S3**. Correlations of neuropsychological tests with second PCA which omits tests that are clinically related to the factors that we identified in the first PCA

|  | Factor 1 | Factor 2 | Factor 3 | Factor 4 |
| --- | --- | --- | --- | --- |
| NonWord repetition Immediate | 0.848** | 0.059 | 0.188 | 0.040 |
| Synonym Judgment | 0.415* | 0.419* | 0.238 | 0.377* |
| Brixton Spatial Anticipation | 0.248 | 0.311 | 0.084 | 0.368* |
| Backward digit span | 0.478** | 0.209 | 0.350^+^ | 0.141 |

Statistical thresholds: ** *p*<0.001, * *p*<0.01, + *p*<0.02

**Supplementary Table S4.** The left brain hemisphere peak co-ordinates with anatomical localisations for our neuroscience results

|  | ***FWE*** | ***FDR*** | ***k*** | ***T*** | ***Z*** | **Coordinates** | | | **Anatomy** |
| --- | --- | --- | --- | --- | --- | --- | --- | --- | --- |
|  |  |  |  |  |  | **x** | **y** | **z** |  |
| ***Dell model parameters, corrected for lesion volume, age, education*** | | | | | | | | | |
| ***s* weight** | 0.024 | 0.005 | 971 | 4.68 | 4.16 | -60 | -8 | -22 | Middle temporal gyrus, anterior |
|  |  |  |  | 3.80 | 3.49 | -62 | -24 | 2 | Middle/superior temporal gyrus, posterior |
|  |  |  |  | 3.38 | 3.15 | -42 | -12 | -18 | Inferior longitudinal fascicle |
| ***p* weight** | 0.003 | 0.007 | 1485 | 4.28 | 3.86 | -50 | -8 | 6 | Heschl’s gyrus/Central opercular cortex |
|  |  |  |  | 3.31 | 3.10 | -60 | -2 | -26 | Middle temporal gyrus, anterior |
|  |  |  |  | 3.30 | 3.08 | -48 | -8 | -16 | Middle/superior temporal gyrus, anterior |
|  |  |  |  | 3.28 | 3.07 | -58 | 2 | 32 | Precentral gyrus |
|  |  |  |  | 3.17 | 2.98 | -62 | -12 | 6 | Superior temporal gyrus/Planum temporale |
|  |  |  |  | 3.02 | 2.85 | -50 | 0 | -14 | Superior temporal gyrus, anterior |
| ***Omissions, corrected for lesion volume, age, education*** | | | | | | | | | |
| **Omission errors** | <0.001 | <0.001 | 2270 | 4.40 | 3.96 | -36 | -6 | -26 | Insular |
|  |  |  |  | 4.02 | 3.67 | -54 | 0 | -26 | Middle temporal gyrus, anterior |
|  |  |  |  | 3.97 | 3.63 | -58 | -12 | -20 | Middle temporal gyrus, posterior |
|  |  |  |  | 3.74 | 3.45 | -38 | -36 | -4 | Inferior frontal occipital fascicle |
|  |  |  |  | 3.69 | 3.41 | -64 | -20 | -14 | Middle temporal gyrus, posterior |
|  |  |  |  | 3.69 | 3.41 | -48 | 2 | -20 | Inferior longitudinal fascicle |
|  |  |  |  | 3.46 | 3.23 | -48 | 14 | -24 | Superior temporal pole |
|  |  |  |  | 3.46 | 3.22 | -44 | 16 | -28 | Middle temporal pole |
|  |  |  |  | 3.38 | 3.16 | -40 | 10 | -28 | Middle temporal pole |
|  |  |  |  | 3.37 | 3.15 | -38 | 10 | -38 | Inferior temporal gyrus, anterior |
|  |  |  |  | 2.95 | 2.79 | -54 | -6 | -4 | Superior temporal gyrus, anterior |
| ***Factor (F) 1-4 in the Omnibus PCA, corrected for lesion volume, age, education*** | | | | | | | | | |
| **F1** | <0.001 | <0.001 | 2562 | 4.31 | 3.87 | -52 | -6 | 6 | Central opercular cortex/ Heschl’s gyrus |
|  |  |  |  | 4.15 | 3.74 | -56 | 0 | 32 | Precentral gyrus |
|  |  |  |  | 4.07 | 3.69 | -54 | 0 | 6 | Central opercular cortex/ Rolandic operculum |
|  |  |  |  | 3.94 | 3.59 | -50 | -4 | -16 | Superior temporal gyrus, anterior |
|  |  |  |  | 3.80 | 3.48 | -60 | -2 | -26 | Middle temporal gyrus, anterior |
|  |  |  |  | 3.76 | 3.45 | -56 | 2 | -36 | Temporal pole |
|  |  |  |  | 3.50 | 3.24 | -60 | -16 | -14 | Middle temporal gyrus, posterior |
|  |  |  |  | 3.49 | 3.23 | -56 | 6 | 16 | Precentral gyrus/ inferior frontal gyrus pars opercularis |
|  |  |  |  | 3.21 | 3.00 | -60 | 12 | -12 | Temporal pole |
|  |  |  |  | 3.16 | 2.96 | -58 | -14 | 4 | Planum temporale |
|  |  |  |  | 3.02 | 2.84 | -50 | 10 | -42 | Temporal pole |
|  |  |  |  | 2.98 | 2.81 | -46 | 8 | 34 | Middle frontal gyrus |
| **F2** | 0.71 | 0.50 | 244 | 3.66 | 3.36 | -58 | -6 | -26 | Middle temporal gyrus, anterior |
|  |  |  |  | 3.39 | 3.15 | -48 | 14 | -24 | Inferior temporal gyrus, posterior |
|  |  |  |  | 3.36 | 3.12 | -40 | -8 | -22 | Inferior longitudinal fascicle |
| **F3** | 0.00 | 0.00 | 5856 | 6.17 | 5.10 | -42 | -46 | 12 | Superior longitudinal fascicle |
|  |  | |  | 5.31 | 4.56 | -40 | -32 | 2 | Inferior longitudinal fascicle |
|  |  |  |  | 4.72 | 4.16 | -46 | -18 | -2 | Planum polare |
|  |  |  |  | 4.47 | 3.98 | -60 | -46 | 14 | Supramarginal gyrus, posterior |
|  |  |  |  | 4.36 | 3.90 | -54 | -60 | 18 | Angular gyrus |
|  |  |  |  | 4.36 | 3.90 | -54 | -26 | 0 | Superior temporal gyrus, posterior |
|  |  |  |  | 4.11 | 3.72 | -60 | -30 | 8 | Planum temporale |
|  |  |  |  | 3.99 | 3.62 | -50 | -48 | -16 | Inferior temporal gyrus |
|  |  |  |  | 3.90 | 3.55 | -68 | -28 | 6 | Superior temporal gyrus, posterior |
|  |  |  |  | 3.31 | 3.08 | -60 | -16 | -14 | Middle temporal gyrus, posterior |
|  |  |  |  | 3.04 | 2.85 | -66 | -54 | 2 | Middle temporal gyrus |
| **F4** | No significant clusters | | |  |  |  |  |  |  |

Significant clusters thresholded at *p*=0.005 voxel height, FWE-cluster corrected *p*<0.05 (except Factor 2 which is at *p*=0.005 voxel height uncorrected, *k*=100). Factor F1 = Phonological ability; F2 = Semantic ability; F3 = Auditory working memory; F4 = Executive-demand.
